# Enantioselectivity in Drug Pharmacokinetics and Toxicity: Pharmacological Relevance and Analytical Methods

**DOI:** 10.3390/molecules26113113

**Published:** 2021-05-23

**Authors:** Maria Miguel Coelho, Carla Fernandes, Fernando Remião, Maria Elizabeth Tiritan

**Affiliations:** 1 Laboratório de Química Orgânica e Farmacêutica, Departamento de Ciências Químicas, Faculdade de Farmácia da Universidade do Porto, Rua Jorge de Viterbo Ferreira, 228, 4050-313 Porto, Portugal; mariamiguelcoelho_2012@hotmail.com (M.M.C.); cfernandes@ff.up.pt (C.F.); 2 Centro Interdisciplinar de Investigação Marinha e Ambiental (CIIMAR), Universidade do Porto, Terminal de Cruzeiros do Porto de Leixões, Avenida General Norton de Matos, s/n, 4450-208 Matosinhos, Portugal; 3 Unidade de Ciências Biomoleculares Aplicadas (UCIBIO)-REQUIMTE, Laboratório de Toxicologia, Departamento de Ciências Biológicas, Faculdade de Farmácia, Universidade do Porto, Rua Jorge Viterbo Ferreira, 228, 4050-313 Porto, Portugal; remiao@ff.up.pt; 4 Instituto de Investigação e Formação Avançada em Ciências e Tecnologias da Saúde, Cooperativa de Ensino Superior Politécnico e Universitário (CESPU), Rua Central de Gandra, 1317, 4585-116 Gandra PRD, Portugal

**Keywords:** chirality, metabolism, enantiomers, chiral analyses, chiral stationary phases

## Abstract

Enzymes, receptors, and other binding molecules in biological processes can recognize enantiomers as different molecular entities, due to their different dissociation constants, leading to diverse responses in biological processes. Enantioselectivity can be observed in drugs pharmacodynamics and in pharmacokinetic (absorption, distribution, metabolism, and excretion), especially in metabolic profile and in toxicity mechanisms. The stereoisomers of a drug can undergo to different metabolic pathways due to different enzyme systems, resulting in different types and/or number of metabolites. The configuration of enantiomers can cause unexpected effects, related to changes as unidirectional or bidirectional inversion that can occur during pharmacokinetic processes. The choice of models for pharmacokinetic studies as well as the subsequent data interpretation must also be aware of genetic factors (such as polymorphic metabolic enzymes), sex, patient age, hepatic diseases, and drug interactions. Therefore, the pharmacokinetics and toxicity of a racemate or an enantiomerically pure drug are not equal and need to be studied. Enantioselective analytical methods are crucial to monitor pharmacokinetic events and for acquisition of accurate data to better understand the role of the stereochemistry in pharmacokinetics and toxicity. The complexity of merging the best enantioseparation conditions with the selected sample matrix and the intended goal of the analysis is a challenge task. The data gathered in this review intend to reinforce the importance of the enantioselectivity in pharmacokinetic processes and reunite innovative enantioselective analytical methods applied in pharmacokinetic studies. An assorted variety of methods are herein briefly discussed.

## 1. Introduction

Chirality is a geometric property related to the absence of symmetry of three-dimensional objects. At the molecular level, this phenomenon is observed in structures with the same connectivity of the atoms but with different spatial arrangement resulting in structures with mirror image of each other (enantiomers). Enzymes, receptors and other binding molecules in biological processes recognize enantiomers as different molecular entities due to their different dissociation constants from the binding sites, leading to different behavior and consequent disparity in pharmacological responses [1,2,3].

More than 50% of the pharmaceuticals on the current market are chiral and administrated as pure enantiomers or as racemate [4]. Interestingly, 20 out of 35 pharmaceuticals approved by the FDA in 2020 are chiral [5], and an increasing trend in authorization requests for chiral drugs has been observed. In recent years, a growing interest in enantiomerically pure substances has been observed in pharmacology and medicinal chemistry, because beyond the advantages of the drug administration as pure enantiomers, toxicity can be associated to the inactive enantiomer [6,7]. The objective of launching enantiomerically pure compounds has been achieved by resorting to the practice of chiral switches or by manufacturing a new single enantiomeric drug [8]. This is a very serious issue, involving research and pharmaceuticals industry concerning the stereochemistry of drugs and its consequences in therapy [1,2,7,9,10]. Thus, the chiral switch technology encompassing the development of single enantiomers, and the revaluation of existing racemates resulted in a better pharmacotherapeutic profile [4]. One of the advantages of chiral switch technology includes a better therapeutic index elaborated by increasing potency and selectivity as well as decreasing side effects. Additionally, there is a more effective and lasting start of the desired effect, combined with a lower propensity to drug interactions, mediated in large part by the exploration of stereoselectivity not only in pharmacodynamics/pharmacokinetics but also in toxicity properties [2,8]. However, despite the unequivocal advantages of the administration of pure enantiomers, many drugs are still commercialize as racemate [9].

Regarding the pharmacokinetics, the associated effects of a drug or its enantiomers in absorption, distribution, metabolism, excretion events, and toxicity (ADME-Tox) are recognized [1]. Stereoselectivity/enantioselectivity can be observed in all pharmacokinetic processes, important and well-known phenomena, mainly in the metabolism of xenobiotics [2].

The enantioselectivity present in drug–protein interactions is considered a determining factor that can interfere in the pharmacokinetic of the enantiomers, with consequences for therapeutic properties and toxicity levels. For example, enantiomers can be absorbed in different proportions when carrier proteins are responsible for drugs passing through cell membranes. There are plasma transport proteins that act in the distribution of both endogenous and exogenous compounds, namely human serum albumin (HSA) and α_1_-acid glycoprotein (AGP). These proteins can also perform other types of functions since drug–protein complexes can restore the concentration of free drugs, which are removed by metabolic elimination processes [10]. During the process of metabolism, differences can be found mainly during first-pass metabolism, where changes in the proportion of plasma enantiomer concentrations can occur when a drug is administered intravenously compared to the oral route. Enantiomers can be metabolized by different enzyme systems, resulting in variations in the rate of metabolic clearance. In addition, factors such as age and sex can also influence the enzyme metabolism of enantiomers [11]. Phenomena related to the inversion of the configuration, such as racemization and enantiomerization, can also occur in metabolism [12]. In fact, metabolic enzymes and membrane transporters influence the entire process of drug absorption, distribution, metabolism, and elimination. Therefore, it becomes difficult and complex to obtain simple answers to questions about the pharmacokinetic profiles of racemate and/or pure enantiomers [2].

Enantioselective analytical methods are crucial to monitor pharmacokinetic studies and to produce accurate data to better understand the role of the stereochemistry in pharmacokinetic processes and the consequent toxicity. Thus, this review describes the importance of the stereoselectivity/enantioselectivity in all stages of pharmacokinetic processes. Furthermore, several enantioselective analytical methods applied to monitor the enantiomers in pharmacokinetic studies are highlighted.

## 2. Enantioselectivity in Drug Pharmacokinetics

### 2.1. Absorption

The absorption of drugs is influenced by their physicochemical properties, the formulation, and the route of drug administration. Cell membranes are biological barriers that selectively influence the passage of drugs [13]. Absorption may favor only one enantiomer or the two enantiomers may differ in their absorption properties [14]. In other words, the diastereoisomeric complexes formed can distinctly modify the biomembrane, consequently leading to different permeability. Enantioselectivity is not expected during passive absorption of enantiomers; however, it may occur when a transport-mediated process is involved [14]. The peptide transporter PEPT1 is one of the most studied drug transporters in the intestine, as it helps to improve the absorption of previously poorly absorbed drugs, which makes it a target molecule [15]. Another well-studied transporter is the proton-coupled folate transporter (PCFT), which is located on the enterocyte membrane and is responsible for mediating folate absorption. PCFT is also able to recognize the methotrexate and aminopterin antifolates, chiral drugs used to treat neoplasia and rheumatoid arthritis. Another example is the organic anion transporter polypeptide (OATP) 2B, which is present in the intestinal border membrane and has the function of capturing chiral compounds such as fexofenadine, celiprolol, and montelukast [15,16].

P-glycoprotein (P-gp) and breast cancer resistance protein are ATP-binding cassette transport proteins that are located in many tissue-barriers, including in the intestinal membrane. These conveyors belong to the efflux pump family and prevent the absorption of drugs in the intestine [15]. P-gp carries various types of drugs, such as anti-cancer agents (responsible for resistance to multiple cancer drugs), antihypertensives, antidepressants, antimicrobials, immunosuppressants, neuroleptics, and opioids, among others [16,17]. Cancer multidrug resistance is one of the main impediments to effective cancer chemotherapy. One of the causes found is due to the increase in the efflux of several chemotherapeutic drugs by the superfamily of the transmembrane transporter of ATP-binding cassettes (ABC), where P-gp is the main highlight. The administration of P-gp drug efflux inhibitors is considered a type of treatment to overcome multidrug resistance in anticancer therapy, blocking the efflux of multiple P-gp-mediated drugs [18].

Despite the importance of enantioselective studies in absorption to understand the pharmacokinetic events, only few examples are described. For example, (*-*)-mefloquine enantiomer (an anti-malarial drug) enantioselective inhibits P-gp, affecting the transport of P-gp substrates, such as cyclosporine (immunosuppressive) and vinblastine (antimitotic) [19]. Fexofenadine is a substrate for the efflux transporter P-gp, for intestinal uptake OATP and for liver uptake transporters OATP1B1, 1B3, and 2B1. Plasma concentrations of (*R*)-(+)-fexofenadine in humans are 1.5 times higher than those of (*S*)-(-)-fexofenadine. The higher plasma concentrations of (*R*)-(+)-fexofenadine are due to the fact that there are multiple transporters capable of chiral discrimination of enantiomers. [20]. A more recent study demonstrated enantioselectivity in assays to assess the permeability of illicit psychoactive drugs methylone and pentedrone (both derived from cathinone) in Caco-2 cells; (*R*)-(-)-pentedrone showed greater permeability and (*S*)-(-)-methylone was the most absorbed enantiomer [21].

### 2.2. Distribution

Distribution is the stage of pharmacokinetics responsible for the reversible transfer of a drug from plasma to tissues. This process goes through two successive phases, starting with the dilution of the drug absorbed in the plasma and then in the distribution of the drug to the peripheral compartments [22].

Enantioselectivity can occur by the different binding of the enantiomers with blood components, such as blood cells and plasma proteins. In most cases, stereospecific binding to these proteins can occur, which can be translated into blood enantioselectivity (or plasma concentration) versus time profiles [23]. In turn, it will be translated into the measured volume of distribution of the two enantiomers and can contribute to the enantioselectivity in clearance. The enantioselectivity related to the interaction with proteins has been widely studied due to three most important proteins present in human plasma, HSA, AGP, and lipoprotein [23]. HSA is the most abundant plasma protein, with plasma being made up of 60% of this protein, while the amount of AGP is very low reaching about only 3% in human plasma [24]. Each drug can interact with more than one protein, and the general plasma proteins binding (PPB) is made by adding each interaction made. PPB causes a limitation of the free movement of the medication, and decreases its volume of distribution, renal excretion, hepatic metabolism, and tissue penetration. In contrast, PPB increases the absorption of the drug and its half-life. Some drugs can vary between interspecies, showing differences in the direction of stereoselectivity in pharmacokinetic processes, including binding to plasma proteins [23].

There are many studies concerning enantioselective binding to HSA [23]. For example, there are drugs such as profens, where the HSA bond is strongest for the (*R*) enantiomer, such as etodolac, indoprofen, ibuprofen, and ketoprofen [25]. It was observed that the different interaction that occurs between HSA with (*R*)- and (*S*)-profens influences metabolic behavior and drug interactions [25]. The preferable interactions of the (*R*)-enantiomer of ibuprofen with HSA causes a greater proportion of the (*S*)-enantiomer in free form. Investigation of the enantioselective interaction of verapamil and amlodipine with HSA showed that (*R*)-(+)-verapamil has stronger bind to HSA compared to (*S*)-(-)-verapamil, while (*S*)-(-)-amlodipine has a stronger bind than the (*R)*-enantiomer [26]. In another study, the binding of (*S*)-omeprazole (esomeprazole) and (*R*)-enantiomer to HSA, under simulated physiological conditions, demonstrated that (*R*)-omeprazole had higher binding constants than (*S*)-omeprazole [27]. Enantioselectivity binding to HSA was also observed for xanthone derivatives [28], a promising class of compounds in medicinal chemistry [29,30].

Acidic drugs tend to bind to HSA, while basic drugs tend to bind to AGP, also known as human orosomucoid (ORM) [22]. The enantioselective binding to the AGP genetic variants ORM 1 and ORM 2 by various anticoagulants demonstrated that all compounds interacted more strongly with the ORM 1 variant than with ORM 2. ORM 1 and native human AGP were the preferred targets for the binding of the (*S*)-enantiomers of warfarin anticoagulants and acenocoumarol [31]. Moreover, it was found out that acenocoumarol possessed the highest enantioselectivity to AGP [31]. The interaction of propafenone enantiomers with human plasma proteins (HSA, AGP) demonstrated the strongest binding to (*S*)-propafenone due to the interaction of this enantiomer with AGP, whereas the binding to HSA was considered weak and non-enantioselective. In addition, drug–drug interaction studies showed that drugs such as verapamil, diazepam, and nifedipine, among others, did not affect the binding of propafenone enantiomers to plasma proteins. The exceptions were quinidine and aprindine, which showed drug interaction in the therapeutic concentration [32]. In another study, the interaction of 13 β-blockers with HSA and AGP was investigated, demonstrating that the strongest interactions occurred between β-blockers and AGP compared to HSA. Additionally, based on the results obtained in this study, both enantiomers showed different binding forces for the same specific site, with (-)-enantiomers showing stronger interactions than (+)-enantiomers. The competition between enantiomers to bind to the same target site may be relevant from a pharmacokinetic point of view when racemates are administered [33].

Regarding the enantioselectivity in membrane transport, studies were carried out on organic cation transporters (OCTs), responsible for transporting hydrophilic cationic substances in the liver and kidneys [34]. A recent study on the interactions of racemic adrenergic drugs with OCTs found that (*R*,*R*)-phenoterol, (*R*,*R*)-formoterol, (*S*)-salbutamol, (*S*)-acebutolol, and (*S*)-atenolol showed greater enantioselective uptake compared to their enantiomers [34].

### 2.3. Metabolism

Metabolism is the pharmacokinetic event where most cases of enantioselectivity occur, since in this stage the presence of a large number of metabolizing enzymes have the capacity to discriminate enantiomers [1]. Due to the three-dimensional nature of the substrate and the chiral recognition by the metabolic enzymes, the drug metabolism may provide many possible pathways for stereoselectivity/enantioselectivity in plasma concentrations. However, the level and direction of stereoselection for a given drug may depend on the path involved [16]. It is important to point out that the stereoselectivity involves not only the substrate’s stereoselectivity, but can also include a second and main aspect, the product’s stereoselectivity [16].

**Figure 1:**
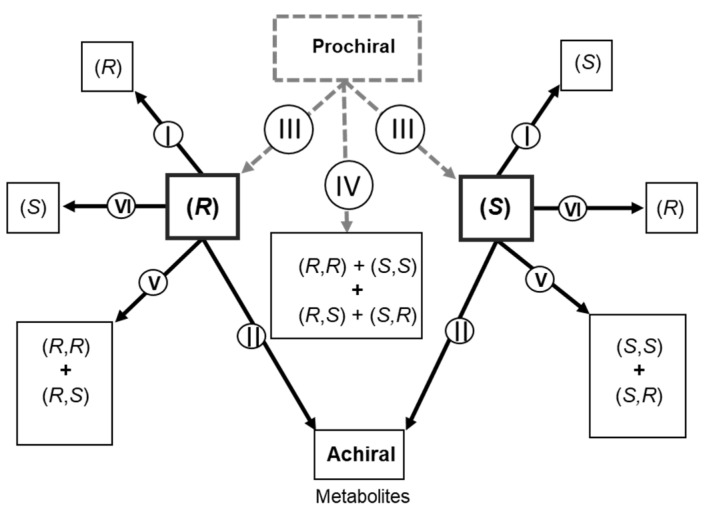
Types of stereoselective metabolism.

According to Prof. B. Testa [2] there are six types of stereoselective metabolism (Figure 1), which cover the vast majority of cases:

Type I: Two enantiomers (*R*) and (*S*) are metabolized in different proportions, leaving the stereogenic element untouched without removing or adding a stereogenic element when forming the metabolite.

Types II: There is a metabolic reaction where the stereogenic element is eliminated, resulting in non-chiral metabolite or one that is more symmetrical. The loss of the chiral property involves a sequence of reactions that results in both pharmacological inactivation and toxification.

Type III: In relation to the product of stereoselectivity, substrates that contain an element of prostereoisomerism are subject to a metabolic reaction that transforms them into products with a stereogenic element, thus occurring the enantioselectivity of the product. However, the chiral metabolites generated through a conjugation reaction with a preformed endogenous chiral molecule cause some concern. As endogenous coupling agents are considered enantiomerically pure, the product does not have stereoselectivity. Other cases can lead to the formation of two diastereoisomeric metabolites from the same pro-chiral substrate containing an element of prodiastereoisomerism.

Type IV: There is only one metabolic reaction that creates two stereogenic centers on a pro-chiral substrate, allowing four stereoisomeric metabolites to be formed.

Types V: A new chirality element is generated with product stereoselectivity ((*R*,*R*)/(*R*,*S*) and (*S*,*S*)/(*S*,*R*)), which differ between the two substrates.

Type VI: A metabolic reaction occurs that is catalyzed by an inversion of the configuration of the stereogenic center.

However, it is known that stereochemistry is such a large field with diverse of reaction mechanisms that it is difficult to categorize all rearrangement reactions and all rare combinations of stereoselectivities that may occur [2].

**Figure 2:**
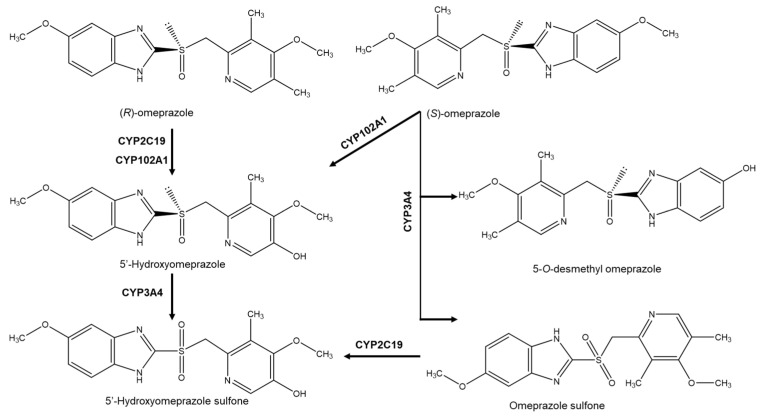
Omeprazole enantiomers metabolism.

In addition, enantioselectivity can occur in pre-systemic metabolism, in the intestine or liver, which can cause differences in the bioavailability of a drug when it is orally administered. This phenomenon is observed particularly in drugs that have a high rate of hepatic metabolism, which can cause different concentrations of enantiomers in blood. The example of omeprazole, which is metabolized by several pathways, including sulfoxidation, demethylation and hydroxylation (Figure 2), shows enantioselectivity in human metabolism [35]. Oxidation of the sulfoxide result in achiral metabolite (sulfone).

Omeprazole is administrated as racemate or enantiomerically pure. However, pharmacokinetic studies suggested that the efficacy of the (*S*)-omeprazole (esomeprazole) as a proton pump inhibitor compared to the (*R*)-omeprazole may be dependent on the metabolic pathway [27]. Metabolism studies have demonstrated enantioselective biotransformation of (*R*)-omeprazole and (*S*)-omeprazole by several known enzymes belonging to the cytochrome P450 (CYP) family, such as CYP2C19, CYP2C9, CYP3A4 and CYP2D6. CYP2C19 primarily metabolizes (*R*)-omeprazole and (*S*)-omeprazole by hydroxylation to hydroxyomeprazole and 5-*O*-desmethylomeprazole, respectively, together with other proton pump inhibitors. On the other hand, CYP3A4 metabolizes (*S*)-omeprazole by oxidation to omeprazole sulfone [27]. The main metabolites of omeprazole found in plasma are hydroxyomeprazole and omeprazole sulfone. The metabolite 5-*O*-desmethylomeprazole that comes from (*S*)-omeprazole has been found and identified in microsomes of human liver. Metabolism studies have shown that esomeprazole is metabolized to a lesser extent compared to (*R*)-omeprazole by CYP2C19. The oxidation of (*S*)-omeprazole by the enzyme CYP3A4 allows less excretion of omeprazole from the human body compared to the hydroxylation pathway [27]. Therefore, the (*S*)-enantiomer has also been marketed as a pure enantiomer, since it has greater bioavailability compared to the racemate and because it results in less intragastric pH variability, proving its greater efficacy in the control of stomach acid secretion. Studies also report that the sale of the (*S*)-enantiomer in pure form has more advantages than the racemate, considering that a small percentage of Caucasian population and 20% of the Eastern population are considered poor metabolizers for the enzyme CYP2C19 [36].

Regarding a type V, related to the product’s stereoselectivity with a new chirality element and the (*R*,*R*)/(*R*,*S*) and (*S*,*S*)/(*S*,*R*) ratios, the bupropion is a good example of a compound with one stereogenic center. After the hydroxylation of the racemic bupropion into hydroxybutpropion (with two stereogenic centers), it is catalyzed only by the cytochrome P450 2B6 (CYP2B6). In humans, bupropion is metabolized in the liver to hydroxybupropion and the aminoketone group is reduced to two amino alcohols (erythro- and threohydrobupropion). Only 1% of the administered dose is excreted unchanged in urine. The human metabolic pathways present stereoselective oxidation, reduction, and glucuronidation of bupropion and its metabolites [37].

**Figure 3:**
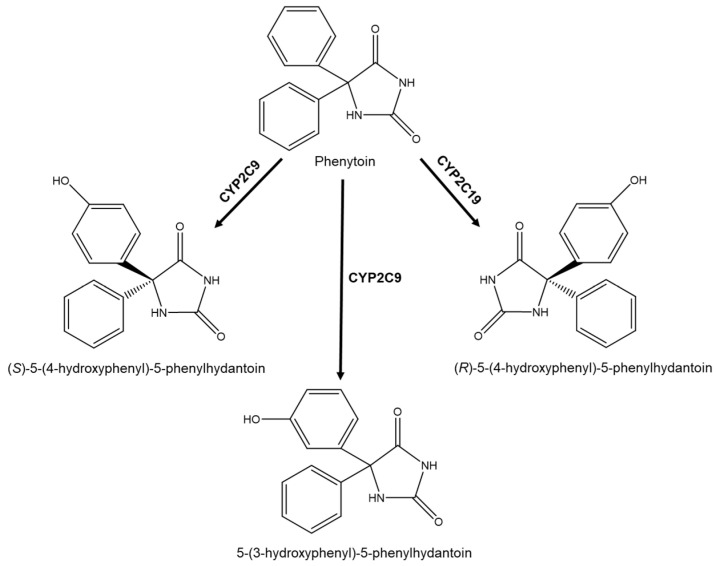
Phenytoin metabolism.

Another interesting case, but related with type III stereoselective metabolism, is the anticonvulsant drug phenytoin (Figure 3), an example of an enantioselective product by aryl oxidation. The molecule is prochiral and its main metabolic pathways pass through the oxidation of phenyl rings to give dihydrodiols, 5-(4-hydroxyphenyl)-5-phenylhydantoin, and 5-(3-hydroxyphenyl)-5-phenylhydantoin. The amounts of 5-(4-hydroxyphenyl)-5-phenylhydantoin produced in humans after phenytoin administration predominated several folds over 5-(3-hydroxyphenyl)-5-phenylhydantoin. Moreover, it also exhibits product enantioselectivity in the reaction of 4-hydroxylation, but not in 3-hydroxylation [38,39,40,41]. Phenytoin’s primary metabolic pathway is hydroxylation by CYP2C9 to 5-(4-hydroxyphenyl)-5-phenylhydantoin; this enzyme is responsible for 95% of the (*S*) configuration. CYP2C19 is responsible for most (*R*) configurations [40].

The structural characteristics of a specific CYP enzyme determine the enantiomeric discrimination associated with the biotransformation of chiral substances; thus, the stereoselectivity present in metabolic reactions can be seen as a characteristic physical property of each enzyme. The formation of a specific metabolite from two enantiomers by a CYP enzyme will allow to categorize and identify the isozyme [22]. It is also known that the enantioselectivity of a metabolizing enzyme can differ from person to person, depending on several factors, such as the genetic variants of metabolizing enzymes [16].

**Figure 4:**
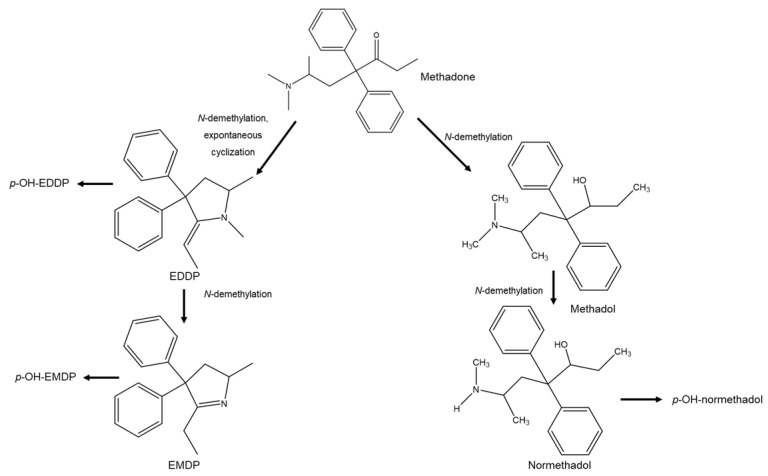
Methadone metabolism. EDDP = 2-ethyl-1,5-dimethyl-3,3-diphenylpyrrolidine; EMDP = 2-ethyl-5-methyl-3,3-diphenyl-1-pyrroline.

Regarding the example of methadone, the concentrations of the (*R*)- and (*S*)-methadone enantiomers when they are above therapeutic levels can cause serious and even fatal side effects. This toxicity can occur differently between individuals due to pharmacogenetics, which influences both the pharmacokinetic and pharmacodynamic properties of the drug. The main isoenzyme responsible for the hepatic metabolism of methadone is CYP2B6, followed by CYP3A4, 2C19, 2D6, and to a lesser extent, CYP2C18, 3A7, 2C8, 2C9, 3A5, and 1A2 [42]. These CYPs metabolize methadone in an enantioselective manner, where CYP2C19, 3A7, and 2C8 favor metabolization of (*R*)-methadone, while CYP2B6, 2D6, and 2C18 prefer (*S*)-methadone and CYP3A4 demonstrated no enantioselectivity. Methadone after hepatic metabolism is eliminated, it undergoes enantioselective *N*-demethylation (Figure 4) to form the main inactive metabolite 2-ethyl-1,5-dimethyl-3,3-diphenylpyrrolidine, which has an elimination half-life with an average of 22 h. This inconsistency in the elimination half-life is also related to pharmacogenetic variability. Due to this variation, the prediction of maximum plasma levels becomes difficult to predict, and may cause plasma concentrations that may be toxic to some individuals. Methadone, clinically used only in racemic form, can be associated with cases of death from overdose due to medical prescriptions of methadone for the treatment of pain [42]. Levomethadone, sold under the brand name *L*-Polamidon^®^, is the (*R*)-enantiomer of methadone. The use of (*R*)-methadone has been demonstrated to be safer than the racemate and is well tolerated [43].

**Figure 5:**
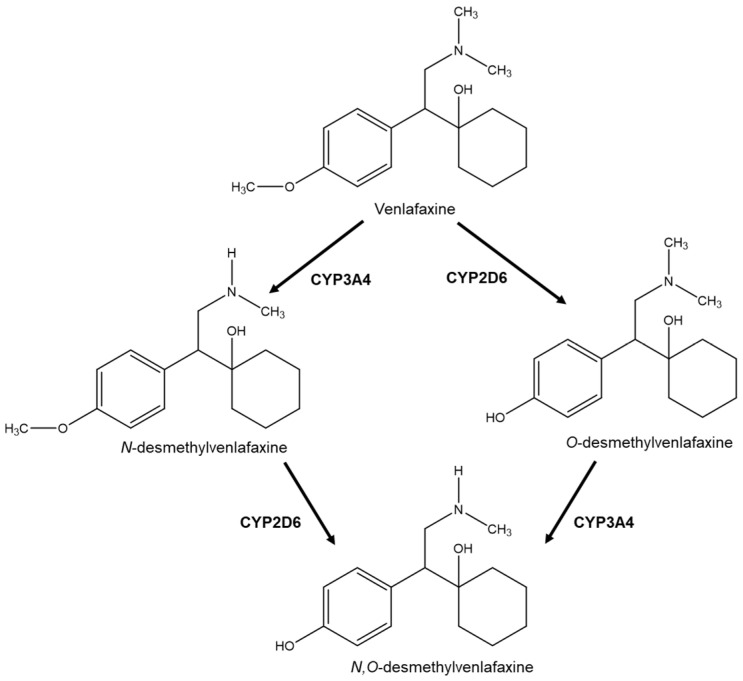
Venlafaxine metabolism.

Another well-studied example concerning enantioselectivity in both the main compound and its metabolites is venlafaxine. Venlafaxine (Figure 5) is a serotonin and norepinephrine reuptake inhibitor marketed in racemic form as an antidepressant agent [44]. However, despite the fact that venlafaxine is marketed as a racemate, the enantiomers show differences in their activity, with the (*R*)-enantiomer inhibiting the synaptic reuptake of norepinephrine and serotonin, while (*S*)-enantiomer inhibits only the serotonin. Both enantiomers undergo hepatic metabolism giving rise to their respective chiral active metabolites (*R*) and (*S*)-*O*-desmethylvenlafaxine metabolized by CYP2D6, (*R*)- and (*S*)-*N*-desmethylvenlafaxine metabolized by CYP3A4 and (*R*)- and (*S*)-*N*,*O*-didesmethylvenlafaxine that come from the metabolization of the other two metabolites by CYP2D6. All metabolites have pharmacological activity, although *N*-desmethylvenlafaxine and *N*,*O*-didesmethylvenlafaxine have lower capacity to inhibit serotonin compared to *O*-desmethylvenlafaxine. CYP2D6 catalyzes the biotransformation of both the enantiomers into *N*,*O*-didesmethylvenlafaxine, although there is greater stereoselectivity for the (*R*)-venlafaxine enantiomer. However, partial metabolic clearance of both enantiomers in *N*-desmethylvenlafaxine, mediated by the enzyme CYP3A4, is not stereoselective [44]. Polymorphisms and drug interactions may occur due to CYP2D6 and, to some extent, CYP2C19, which have been shown to significantly influence the pharmacokinetics and metabolic clearance of venlafaxine and the formation of its active metabolite, *O*-desmethylvenlafaxine [45].

**Figure 6:**
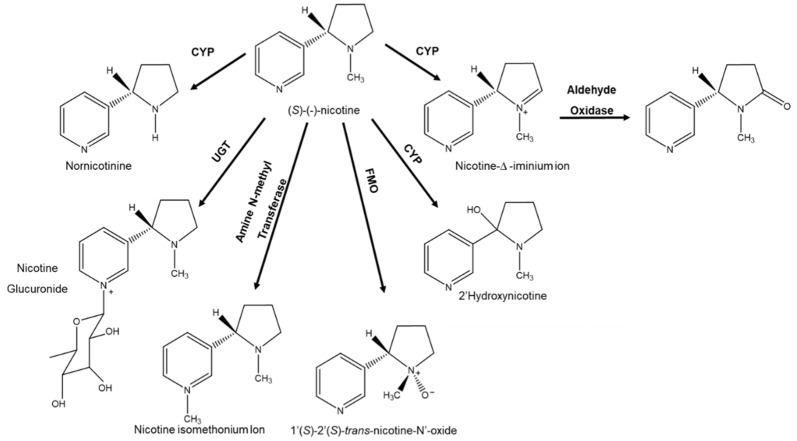
Nicotine metabolism.

Another interesting example is related to the oxygenation reactions of tertiary amines and sulfides, which can generate stable chirality centers depending on the structural characteristics of the substrate. These reactions are catalyzed mainly by flavin-containing monooxygenases (FMOs) or CYPs, as is the case of nicotine (Figure 6) [46]. Natural (*S*)-(-)-nicotine during metabolism undergoes oxygenation of *N* to *N’*-nicotine oxide. In humans, the reaction is catalyzed by FMO3 and shows enantioselectivity of the product forming *N’*-oxide of (1’*S*,2’*S*)-*trans*-nicotine in high and even exclusive amounts (enantiospecificity). The product’s stereoselectivity seems to be less marked in catalysis of other FMOs. In general terms, the stereoselectivity of the product observed is considered dependent on the configuration of the nicotine enantiomer used as a substrate [2].

Differences between species are also common; for example, the metabolic oxidation of felodipine in humans is greater for the (*S*)-enantiomer, whereas rats and dogs preferentially metabolize the (*R*)-enantiomer [47].

Drug metabolism can also be affected by acute or chronic diseases that alter the structure or function of the metabolizing organ. Depending on the severity, in the case of liver diseases, these can alter the metabolism capacity in the liver by causing, for example, a decrease in the synthesis of plasma proteins or enzymes involved in biotransformation reactions and also alteration in the drug binding [48]. Evaluation of the pharmacokinetics of nebivolol enantiomers in patients with chronic kidney disease showed a greater plasma proportion of *L*-nebivolol [49]. Another example is the pharmacokinetics of cyclophosphamide in patients with systemic sclerosis and multiple sclerosis that presented higher plasma concentrations of the (*S*)-(-)-cyclophosphamide enantiomer due to the preferential formation of the (*R*)-4-hydroxycyclophosphamide metabolite [50].

Drug–drug interactions are another factor that can affect the metabolism. Considering the racemic praziquantel (PZQ), the drug of choice for the treatment of schistosomiasis, the metabolism of (*R*)-PZQ was mainly catalyzed by CYP1A2 and CYP2C19, whereas the metabolism of (*S*)-PZQ was mainly by CYP2C19 and CYP3A4. CYP3A4 was estimated to contribute 89.88% to metabolism of (*S*)-PZQ. Therefore, any variation that may occur at the enzymatic level responsible for the metabolism of PZQ is likely to have a major impact on the pharmacokinetics of the drug. PZQ metabolism may vary due to drug interactions caused by the inhibition or induction of CYPs 1A2, 2C9, 2C19, and 3A4. A recent study on the drug–drug interaction with ketoconazole, a potent inhibitor of CYP3A, showed differences in the bioavailability of PZQ enantiomers in human plasma and the mean clearance was higher for (*R*)-PZQ as compared to (*S*)-PZQ. However with the co-administration of ketoconazole, the clearance of (S)-PZQ reduced by a third, whereas the clearance of (*R*)-PZQ slightly increased [51].

### 2.4. Excretion

Excretion is the last phase of pharmacokinetics, in which a drug and/or its metabolites are eliminated. The main excretory processes involve bile and urine, by liver and kidney, respectively. It is possible to verify stereoselectivity in clearance and plasma concentrations in both biliary and urinary excretory processes [16]. In general, hydrophilic compounds have a higher and faster percentage of excretion than lipophilic compounds [16,22].

Renal excretion consists of three processes: glomerular filtration, active tubular excretion, and passive tubular reabsorption. Glomerular filtration is the process responsible for the elimination of the drug or metabolites in free form but this process is not stereoselective. Tubular excretion is the process where ionized compounds are excreted from the tubules by specific carriers. This process is considered mainly stereoselective due to the different interactions that the enantiomers present with the transporters. Plasma pH is the main factor that influences the degree of ionization of a compound, thus playing an important role in this process. The total renal elimination of a drug and its metabolites, in general, is considered a stereoselective event [22]. However, data about enantioselectivity renal excretion are scarce. A study focused on the excretion of albendazole sulfoxide and albendazole sulfone enantiomers in the urinary tract in individuals with neurocysticercosis found that there was enantioselectivity in the renal excretion of albendazole sulfoxide as a complementary mechanism to the metabolism responsible for plasma accumulation of (+)-albendazole sulfoxide [52]. It is important to highlight that most reported enantioselective excretion are a consequence of metabolism. The example concerns 3,4-methylenedioxy-methamphetamine (MDMA), usually consumed as racemate, enantioselectivity studies showed that during the first hours after racemic ingestion of MDMA, the (*S*)-enantiomer is the one that has a higher and faster rate of elimination from plasma compared to the (*R*)-enantiomer. Enantioselective metabolism is the most likely cause for explaining the different pharmacokinetics of the MDMA enantiomer in humans [53]. Thus, the fact that it seems that there is stereoselectivity in excretion does not necessarily reflect that there is stereoselectivity during the secretion process itself. For example, in the case of bisoprolol, it is eliminated through hepatic and renal processes. After oral administration of bisoprolol in the form of racemate, the cumulative recovery of (*S*)-bisoprolol in urine was found to be significantly greater than that of (*R*)-bisoprolol. However, no differences were found in the renal clearance of both enantiomers, which can be explained by the difference during metabolic clearance [54]

## 3. Toxicity

Chiral drugs exhibit stereoselective behavior at all stages of the aforementioned disposal processes, thus leading to complex pharmacological and toxicological behaviors. There are three categories where chiral drugs can be inserted and compared in terms of the enantioselectivity toxicological profile: toxicity in distomers (the less active enantiomer), in eutomers (the more active enantiomer), and in racemates [19]. Drugs can also be converted into active metabolites by the metabolic pathway in a living system. Generally, pharmacologically active metabolites have similar pharmacological effects to the parent compound, but can exhibit other types of unwanted activities that can lead to clinical toxicities. The covalent modification of proteins caused by drugs and/or their metabolites can lead to the appearance of toxic effects. It is common to associate this toxicity with overdoses, drug interactions, and adverse effects in therapeutic doses. Several therapeutic agents have already demonstrated stereospecific toxicological effects [19].

The mechanisms of drug-induced toxicity are generally complicated, as multiple factors can be associated, such as toxicity based on the target mechanism, creation of hypersensitivity, immunological reactions, pharmacological effect outside the target organ, biological activation of toxic metabolites, or idiosyncratic toxicities [22].

It should be noted that many chiral drugs have toxicity associated with distomers [19]. For example, ofloxacin (racemate used to treat bacterial infections) exhibits high antimicrobial activity by the (*S*)-enantiomer (eutomer). One of the effects of ofloxacin is the possibility of causing neurotoxicity, which is believed to reside in the (*R*)-enantiomer due to stereospecific effects. For this reason, the ofloxacin is also marketed in pure enantiomeric form as levofloxacin, the (*S*)-enantiomer [55]. There are other examples, such as ketamine, in which the (*S*)-enantiomer is responsible for the anesthetic and analgesic effects in both animals and humans, whereas the (*R*)-enantiomer of ketamine (distomer) leads to reactions such as hallucinations, agitation, and restlessness [56,57]. Interestingly, esketamine, the (*S*)-enantiomer, has been recently approved by the FDA for treatment-resistant depression [58]. However, another recently study indicated that the arketamine, (*R*)-enantiomer, might produce fast-onset and sustained antidepressant effects in treatment-resistant depression patients with a favorable safety profile [59].

Pharmacological and toxicological effects can coexist in the same enantiomer or in both. For example, the pharmacological profiles of citalopram eutomer (escitalopram) and its metabolite *N*-desmethylcitalopram are very similar to the racemate profile. However, after the administration of escitalopram an inter- and intra-patient variability in the concentrations in the serum have been observed. As it is a serotonergic agent, escitalopram can cause adverse reactions, such as hyponatremia, serotonin syndrome, and restless legs syndrome. In conclusion, the clinical superiority of escitalopram is yet to be demonstrated [60].

Another known case concerns albuterol, used for the treatment of asthma, in which the (*R*)-enantiomer is the eutomer and (*S*) the distomer. When interacting with β_2_-adrenoceptors, (*R*)-albuterol has bronchodilation and it is bronchoprotective, whereas (*S*)-albuterol has no activity towards β_2_-adrenoceptors. For many years, the distomer was presumed to have no biological activity. However, it has been found that regular and excessive use of albuterol can induce paradoxical reactions in some patients with asthma [61]. As activation of β2-adrenergic receptors could not be associated with these effects, the pharmacological profile of (S)-albuterol was studied with more attention, verifying that (*S*)-albuterol intensifies bronchoconstrictor responses and induces hypersensitivity of airways of asthmatic patients. These reactions that come from the albuterol distomer may explain the increase in allergic bronchospasm caused by racemic albuterol, due to the activation of eosinophils in the asthmatic airways. As (S)-albuterol is metabolized slowly and retained in the airways, these paradoxical effects become higher during regular and excessive use of racemic albuterol, and consequently, the eutomer (levalbuterol) started to be commercialized since it has advantages over racemate in asthma therapy [61].

Currently, pharmacokinetic studies based on achiral compounds are not adequate to describe the fate of chiral drugs. The study of chiral drug enantioselectivity/stereoselectivity is important to provide an explanation of the effect–concentration relationships in plasma, urine, and even feces, which should not be based on the total concentration of the drug. Due to improvements in the sensitivity and selectivity of biological assays, together with developments in chiral analysis, the studies of the enantioselective drug disposition have been accomplished [62].

## 4. Chiral Analysis in Pharmacokinetics

To follow the enantioselective behavior in ADME-Tox, a suitable analytical method is needed. Chiral bioanalytical assays are important to study the different concentrations of individual enantiomers and/or their chiral metabolites in biological samples, in order to evaluate all pharmacokinetic events [62,63]. In addition, chiral analysis are crucial to assess the presence of chiral inversion or racemization caused during the biotransformation process [12,64]. Along with the development of novel and simple sample preparation procedures, chromatography is the main analytical technique used nowadays. A recent review covered current methods and applications in chiral analysis in biological samples for clinical and forensic research [62]. The sample preparation processes, the analysis by chromatography (gas (GC) and liquid (LC)), capillary electrophoresis, and supercritical fluid chromatography (SFC) with a diversity of detectors have been presented and discussed in a previous study [62]. Bioanalysis by capillary electromigration (CE) methods have also been recently revised [65]. The CE technique is a technique of high speed, resolution, and sensitivity and lower detection limits. It is also considered of low cost when compared to chromatographic methods. Despite the extensive application for research purposes, this technology has not yet been applied to analyses in the routine arena. Reasons for this might be the lack of knowledge about this technology, difficulty in establishing a method with a mass spectrometer (MS) detector, and the low distribution of CE-MS equipments [65]. In spite of the application of the diversity of methods for bioanalysis, LC with mass analyzers has become the technique of choice due to its versatility, high selectivity, sensitivity, and the low quantification limits achieved [66]. Moreover, due to the numerous commercially available chiral stationary phases (CSPs) and the high versatility provided by the multimodal elution conditions, LC has been the most applied technique to quantify enantiomers and to determine the enantiomeric ratio [67,68], for application in pharmaceutical preparations and in biological samples for therapeutic and toxicological purposes [69]. Regardless, the hard task of new applications relies on the right selection of a CSP and mobile phase conditions, mostly achieved by trial and error, especially considering that in most cases the detection by MS is mandatory and sets a restriction on mobile phase conditions for the enantioseparation. Due to the complexity of the search for different CSPs and elution conditions, systematic workflow for chiral method development has been developed [70]. Predictive models capable of estimating the enantioselectivity of a given drug in different chiral selectors have been published, showingto be a useful strategy for chiral column selection [71,72]. The type of matrix and the clean-up also play an important role in the method development. The trend in innovation is towards reaching higher levels of detectability and lower sample volumes with feasible applicability. A selection of enantioselective methods applied in pharmacokinetic studies are presented in Table 1. Most studies reported plasma as the matrix of choice; only a few of them consider the identification of new metabolites, the most difficult task in pharmacokinetic evaluation.

The analytical methods to monitor the pharmacokinetics of local anesthetics were recently reviewed [6]. The changes in the pure enantiomers for different anesthetics, such as bupivacaine, mepivacaine, prilocaine, and ropivacaine, were studied. For the enantioselective separation of racemates, chromatographic and electrophoretic methods were used. In the case of LC, several CSPs based on cyclodextrins, macrocyclic antibiotics, or substituted carbamates from cellulose and amylose were tested. It was concluded that although the use of local anesthetics in an enantiomerically pure form has advantageous properties in terms of its activity and lowtoxicity, its potential has not yet been fully studied and verified [6]. The enantioselective methods to study pharmacokinetics and pharmaceutical formulations of venlafaxine were also recently revised, which encompass LC-UV, LC-MS, LC-MS/MS, and capillary electrophoresis methods [44]. Many case studies were reported in this review, such as a study on healthy Chinese volunteers, in whom, after the administration of venlafaxine, (*S*)-venlafaxine concentrations were approximately twice as high as those of (*R*)-venlafaxine, strongly suggesting a stereoselective disposition [103]. A chiral LC-MS/MS method using a Chirobiotic^®^ V column operating in the multiple reaction monitoring allowed an LOQ of 0.28 ng/mL. The LC-MS/MS method permitted the determination of venlafaxine enantiomers in plasma and provides complete and reliable measurements of the pharmacokinetics of the two enantiomers. It was suggested that the (*R*)-venlafaxine was metabolized faster than (*S*)-venlafaxine [103].

Antihistamine drugs, such as cetirizine, among others, are used to relieve or prevent symptoms of hay fever and allergies. For example, in the case of cetirizine, used to treat urticaria, the (*R*)-cetirizine is the enantiomer pharmacologically active, while the (*S*)-cetirizine is inactive [104]. Many methods have already been applied for chiral analysis of antihistamines, such as CE, SFC, and LC. Among them, LC-MS using the Chiralpak^®^ IC column was recommended [104].

The use of CSPs based on proteins for LC analysis has been of special interest due to their unique stereoselectivity properties being successfully applied for the chiral resolution of compounds and also because they comprise biological proteins, used for bioaffinity studies [105]. CSPs comprising immobilized HSA have been used for the enantioresolution of drugs mainly with neutral and acid character [10] to relevant information regarding the transport in the body [28]. Several studies reported that HSA-CSPs were efficiently used for enantioseparation of chiral analytes and the chromatographic results reflected their binding to HSA in vivo [106,107]. HSA exhibits a broad affinity and chiral recognition for a variety of classes of chiral drugs due to its flexible structure comprising multiple binding sites [108,109].

One of the major challenges in bioanalytical methods is the fact that chiral metabolites can have different chemical properties. The pharmacokinetics, including metabolism, of linagliptin was investigated in healthy volunteers. Linagliptin, in unaltered form, was the present in all the matrices investigated. After oral administration, the main metabolite 7-but-2-ynyl-8-(3*S*-hydroxy-piperidin-1-yl)-3-methyl-1-(4-methyl-quinazolin-2-ylmethyl)-3,7-dihydro-purine -2,6-dione (CD1790) was observed with high more than 10% of parent compound systemic exposure. The metabolite was identified as (*S*)-3-hydroxypiperidine. The enantiomer of linagliptin and CD1790 were not observed with suitable enantioselective LC-MS methods using a Chiralpak^®^ IA column. In conclusion, the metabolism has only minor contribution to the overall disposition and elimination in humans. Linagliptin was mainly eliminated unchanged in feces and renal excretion was low [102].

A combination of achiral and chiral stationary phases may be used to add the additional separation power. Two-dimensional chromatography (2D) also allows the possibility of reducing the total chromatographic run time by allowing the simultaneous of achiral and chiral chromatographic separation from two consecutive injections. The achiral column is always placed in front of the chiral column using either a heart-cut column switch or direct front or back elution. The achiral column also serves as the guard column to protect the chiral column or to clean up the sample. This also allows to trap the late eluting interference on the achiral column, subsequently being removed using back-flushing or gradient elution. For example, a heart-cutting 2D-LC method to quantify the enantiomers of propranolol (recently expanded to treat pediatric tumors) and their metabolites was successfully developed and optimized. The (*S*)-propranolol and (*R*)-propranolol, and its hydroxy metabolites, namely the isomeric (*S*)-4’-hydroxy propranolol, (*R*)-4’-hydroxy propranolol, (*S*)-5’-hydroxy propranolol, (*R*)-5’-hydroxy propranolol, (*S*)-7’-hydroxy propranolol, and (*R*)-7’-hydroxy propranolol, were discriminated and quantified in one chromatographic run. Highly sensitive hyphenated mass detectors for 2D-LC allow the detection of drugs and their metabolites. The enantiomers of propranolol and its metabolites were separated and quantified in human urine samples, allowing for a calculation of the excretion rates [78]. Another 2D LC-MS/MS was used for the simultaneous quantification of fluoxetine and norfluoxetine enantiomers in biological samples (human milk) using direct sample injection. In the first dimension, an octadecyl column of bovine serum albumin was used for the depletion of milk proteins and the Chirobiotic^TM^ V2 column was used for enantiomeric separation in the second dimension. In general, the average concentration found of (*S*)-norfluoxetine was 2.5 times higher than (*R*)-norfluoxetine, while the average concentration of (*S*)-fluoxetine was also 2.3 times higher than (*R*)-fluoxetine. The average concentrations of (*S*)-norfluoxetine was 1.4 times higher than (*S*)-fluoxetine [101]. The 2D-LC-MS/MS method with achiral and chiral separation was used as an essential tool for innovation in this field.

## 5. Conclusions

Enantioselectivity can be verified in all phenomena of pharmacokinetics and needs an enantioselective analytical method to ensure the safe use of chiral drugs. Pharmacokinetic data of chiral drugs obtained from non-stereoselective methodologies can be highly misleading with regards to the pharmacological/toxicological effects or therapeutic benefit due to the inaccurate methods to measure the concentration of both enantiomers separately. The advances in enantioselective studies have a remarkable contribution in the current tendency for the administration of pure enantiomers instead of a racemates. The advantages of administration of pure enantiomers, including safety, are undeniable. The enantioselective studies of the pharmacokinetic events allow to understand whether the enantiomers have the same or a different pathway in the pharmacokinetic process, especially with respect to metabolism, and can be used to consider the possibility of associated toxicity.

Methodologies that allow the enantioseparation of drugs to better understand the pharmacokinetic events and toxicological mechanisms are well established but still need improvement in in vivo studies in human matrices and also in the development of methods for further routine applications. Enantioselective analytical methods by LC using CSPs is the primary choice to quantify enantiomers in biological matrices. Despite the use of LC-DAD, LC-UV and LC-FD, the use of LC-MS/MS allows to obtain a better selectivity, sensitivity, and unambiguous identification of the target substances. Nevertheless, innovation in sample preparation to attain a faster and more efficient chiral separation methods is still needed.

## Figures and Tables

**Table 1:** Enantioselectivity analytical methods in biological matrices for the pharmacokinetics profile.

Compound	Matrix	Enantioselectivity in Pharmacokinetics	Analytical Method	Observation	Ref.
***R*** **/*S* (±)-tramadol** **(TMD)**	Human Plasma	The main metabolic pathways are *O*-demethylation in *O*-desmethyl tramadol (1st metabolite) by the polymorphic cytochrome isoenzyme P450 2D6 (CYP2D6) and *N*-demethylation for *N*-desmethyl tramadol (2nd metabolite) by CYP2B6 and CYP3A4.	LC-FD; Column: Chirapak^®^ AGP Mobile Phase: 30 mM diammonium hydrogen phosphate buffer: ACN (98.9:1:0.1, *v*/*v*) (pH 7) Flow rates: 0.5 mL min^−1^	(+)-TMD has greater affinity for µ receptor and inhibits serotonin reuptake, while the (-)-TMD is a more effective inhibitor of norepinephrine reuptake.	[73]
**nadolol** **(NAD)**	Human Plasma	NAD is almost exclusively excreted unchanged in urine. As the degree of stereoselectivity in renal clearance is low, a decrease in renal function can be expected to cause proportionally equal increases in plasma concentrations of four enantiomers.	LC-UV and LC-FD; Column: Chirapak^®^ AD-H Mobile Phase: HEX: EtOH: DEA:Trifluoroacetic (88:12:0.4:0.23 *v*/*v*/*v*/*v*) Flow rates: 1.0 mL min^−1^	Only one of the eight stereoisomers (*R*,*S*,*R*-NAD) is responsible for the therapeutic effect. The main challenges encountered when employing the CSP to analyze biological samples is the co-elution of enantiomers of interest and interfering matrix compounds.	[74,75]
***R*** **/*S* (±)-ketamine** **(KET)**	Rat Plasma	(*S*)-KET is metabolized to (*S*)-norketamine, which produces rapid and sustained antidepressant-like effects and could be an alternative to (*S*)-KET.	LC-MS/MS Column: Chiralpak^®^ AS-3R Mobile Phase: 1 mM ammonium bicarbonate: ACN (54:46 *v*/*v*) Flow rates: 1.0 mL min^−1^	(*R*)-KET had greater potency and longer lasting antidepressant effects than (*S*)-KET. However, (*R*)-KET has fewer detrimental side effects than either (*R*,*S*)-KET or (*S*)-KET.	[76,77]
***R*** **/*S* (±)-propranolol** **(PHO)**	Human Urine	90% of ingested PHO was found in 12 metabolites in urine.	2D-LC-MS/MS and UV; Column: PhenylHexyl in the first dimension. Teicoplanin-based chiral column in the second dimension. Mobile phase: 10 mM ammonium formate in water (adjusted to pH 3): MeOH (gradient mode). Flow rate: 0.4 mL min^−1^ and the column temperature were 30 °C (first dimension).	The (*S*)-PHO shows more β-blocking activity than (*R*)-PHO.	[78]
***R/S*** **(±)-salbutamol** **(SBT)**	Human Urine	(*R*)-SBT l is metabolized up to 12 times faster than (*S*)-SBT. Both enantiomers are actively excreted in urine.	HPLC-ESI-MS; Column: Chirobiotic^®^ V Mobile phase: MeOH: AcOH: TEA (100:0.025:0.75 *v*/*v*/*v*)	(*R*)-SBT is 80 times more active than (*S*)-SBT. The inactive (*S*)-SBT may have undesirable actions on lung function.	[79,80]
***R/S*** **(±)-omeprazole** **(OMZ)**	Human Plasma	The pharmacokinetic studies suggest that the efficacy of (*S*)-enantiomer depends on the metabolic pathway and excretion.	LC-UV; Column: Lux^®^ Amylose-3 Mobile phase: HEX: EtOH (70:30 *v*/*v*) Flow rate: 1.0 mL min^−1^	The clearance of (*S*)-OMZ is lower than that of the (*R*)-OMZ.	[27,81]
***R/S*** **(±)-venlafaxine** **(VNF)**	Rat Plasma	VNF is metabolized by CYP450 enzymes and the most abundant metabolite is *O*-desmethylvenlafaxine, found in plasma in high concentrations.	LC-MS/MS; Column: Chirobiotic^®^ V Mobile phase: 30 mM AA, pH 6.0: MeOH (15:85)	The (*S*)-VNF inhibits serotonin reuptake, while the (*R*)-VNF inhibits serotonin and norepinephrine reuptake. *O*-desmethylvenlafaxine is a pharmacologically active metabolite which contributes to the therapeutic effect of VNF.	[44,82]
***R/S*** **(±)-ibuprofen** **(IBU)**	Human Plasma	There is a conversion of the inactive (*R*)-enantiomer to its pharmacologically active (*S*)-enantiomer.	LC-MS/MS; Column: LUX^®^ Cellulose-3 Mobile phase: 0.05% FA solution: MeOH (30: 70 *v*/*v*) Flow rate: 0.2 mL min^−1^	(*S*)-IBU has been reported to be 160 times more active that (*R*)-IBU.	[83]
***R/S*** **(±)-citalopram** **(CIT)**	Human Plasma and breast milk	The (*S*)-CIT enantiomer and its ((*S*)-DCIT and (*S*)-DDCIT) metabolites are eliminated more quickly than their enantiomers.	LC-UV; Column: Phenomenex^®^ Lux Cellulose-2 Mobile phase: AA (pH 9.0; 20 mM): ACN (gradient mode) Flow rate: 0.6 mL min^−1^	In the treatment of depression, (*S*)-CIT is over 100-fold more potent as a selective serotonin reuptake inhibitor than (*R*)-CIT.	[84,85]
***R/S*** **(±)-verapamil** **(VRP)**	Human Plasma	After intravenous administration, the plasma clearance and apparent volume of distribution of (*S*)-VRP are almost twice as high as those of (*R*)-VRP.	LC-FD; Column: Chiralpak^®^ AD Mobile phase: HEX: ISO: EtOH (85:7.5:7.5 *v/v*) and 0.1% TEA Flow rate: 1.5 mL min^−1^ with column oven temperature was 30 °C	(*R*)-(+)-VRP has far less cardiotoxicity than (*S*)*-*(-)VRP. However, the pharmacological potency of (*S*)-VRP is 10–20 times greater than its (*R*)-VRP in terms of negative chromotropic effect on atri-ventricular conduction and vasodilatator in man.	[55,86]
***R/S*** **(±)-felodipine** **(FLP)**	Human Plasma	The elimination of FLP from the body depends on the metabolic clearance of CYP450. The metabolism rate of (*R*)-FLP was faster than that of (*S*)-FLP in human liver microsomes.	LC-UV Column: Chiracel^®^ OJ Mobile phase: HEX:ISO (5:1 *v*/*v*) Flow rate: 1.0 mL min^−1^ with column oven temperature was 40 °C	(*S*)-FLP possesses the ability to antagonize the calcium channels, assuming no (inter)activity of the (*R*)-enantiomers.	[47,87]
***R/S*** **(±)-methadone** **(MTD)**	Human Plasma	(*R*)-MTD is less bound to plasma proteins, with AGP being the predominant binding protein. Few studies to date have found the variability in protein binding in the pharmacokinetics of total (*R*)- and (*S*)-MTD.	LC-UV Column: Astec^®^ Cyclobond Type I-Beta RSP Mobile phase: ACN: MeOH (75:25 *v*/*v*) and 1% TEA Flow rate: 0.6 mL min^−1^ with column oven temperature was 18 °C	(*R*)-MTD has been shown to be responsible for most of the analgesic activity. Elevated (*R*)-MTD levels can increase the risk of respiratory depression, while elevated (*S*)-MTD levels can increase the risk of severe cardiac arrhythmias.	[42,88]
***R/S*** **(±)-trelagliptin** **(TLG)**	Dog plasma	The absolute bioavailability of (*R*)-TLG was identified to be 128.2%. No chiral bioconversion of (*R*)-TLG to (*S*)-TLG was observed.	LC-MS/MS Column: Chiralcel^®^ OX-3R Mobile phase: 10 mmol/L ammonium bicarbonate: ACN Flow rate: 0.6 mL min^−1^	(*R*)-TLG is a highly selective and long-acting dipeptidyl peptidase IV inhibitor used for the treatment of type 2 diabetes.	[89]
***R/S*** **(±)-fexofenadine** **(FXF)**	Human Plasma And Urine	The highest plasma concentrations of (*R*)-FXF are attributed to the combination of several carriers capable of chiral discrimination of enantiomers.	LC-MS/MS Column: Chirobiotic^®^ V Mobile phase: MeOH: 7 mM AA, pH 4.25 (97:3 *v*/*v*) Flow rate: 0.7 mL min^−1^	(*S*)-FXF is a more potent human histamine H1 receptor inverse agonist and shows greater receptor occupancy than (*R*)-FXF.	[90]
***R/S*** **(±)-metoprolol** **(MET)**	Human Plasma	Oral bioavailability of (*S*)-MET is lower after administration of the pure (*S*)-enantiomer solution than when the same dose of the (*S*)-form is administered as a racemate.	LC-ESI-MS; Column: Chirobiotic^®^ V Mobile phase: EtOH: MeOH: AcOH (pH 6.7): TEA (50: 50: 0.225: 0.075 *v*/*v*/*v*/*v*)	(*R*)-MET is less effective in reducing the mean arterial blood pressure than (*S*)- and *R*/*S*-MET.	[91,92]
***R/S*** **(±)-amlodipine** **(AML)**	Rat Plasma	AML has a low rate of hepatic excretion and is absorbed by liver tissue, due to high tissue affinity, and only afterwards is it redistributed to the systemic circulation. These properties result in peak plasma concentration and longer plasma clearance.	LC-UV Column: Chiralcel^®^ OZ-RH Mobile phase: ACN: H_2_O (10 mM AA, 0.5% ammonia solution) (95:5 *v*/*v*) Flow rate: 0.5 mL min^−1^	Studies support the conclusion that there is no racemization in vivo. Only (*S*)-AML possesses vasodilating properties.	[93,94]
***R/S*** **(±)-asenapine** **(ASP)**	Rat Plasma	Individual enantiomers of ASP revealed that the (+)-ASP shows a better plasma concentration compared to the (-)-ASP.	HPLC-DAD Column: Lux^®^ Cellulose-1 Mobile phase: ACN: 50 mM ammonium bicarbonate in water (60: 40 *v*/*v*) Flow rate: 0.7 mL min^−1^	Only trans isomers, in the form of an enantiomer racemate *R*, *R* and *S*, and *S*, have been approved due to receptor binding. (*R*,*R*) and (*S*,*S*) enantiomers of ASP block behavioral responses mediated by 5-HT2A, 5-HT2C, 5-HT1A, D2, and D1 receptor ligands. The metabolite 11-*O*-sulfated-asenapine demonstrated an inability to cross the blood–brain barrier.	[95,96,97]
***R/S*** **(±)-nimodipine** **(NMP)**	Human Plasma	(−)-(*S*)-NMP was more rapidly eliminated than the (+)-(*R*) counterpart.	LC-MS/MS Column: (*S*,*S*)-Whelk^®^ O1 Mobile phase: MEOH: H_2_O (75:25 *v*/*v*) Flow rate: 0.1 mL min^−1^	(−)-(*S*)-NMP is approximately twice as potent a vasorelaxant as the racemate.	[98]
***R/S (±)-*** **butoconazole** **(BTZ)**	Rat Plasma and tissues	The concentration of (+)-BTZ was higher than that of (-)-BTZ, indicating that (+)-BTZ tends to exist in various tissues leading to a slower metabolism. The higher concentration of (+)-BTZ in plasma can cause differences in the enantioselective distribution between (-)- and (+)- BTZ.	LC-ESI-MS/MS Column: Chiralpak^®^ IC Mobile phase: ACN: 10mM aqueous AA (90:10 *v*/*v*) Flow rate: 0.6 mL min^−1^	Commercially enantiopure standards for BTZ were not available. The semi-preparative enantioseparation of the butoconazole was obtained by LC-UV. Some azole enantiomers may exhibit distinct differences in the biological activity.	[99]
***R/S*** **(±)-carbinoxamine** **(CAR)**	Rat Plasma	It is currently unclear whether CAR enantiomers have different pharmacodynamic, toxicological, or pharmacokinetic properties. However, stereoselectivity does not occur in absorption and excretion.	LC-MS/MS Column: Chiralpak^®^ ID Mobile phase: ACN: H_2_O: ammonia solution (90:10:0.1 *v*/*v*/*v*). Flow rate: 0.6 mL min^−1^	The method used for enantioseparation of CAR can be applied to other antihistamines, such as meclizine, cloperastine, azelastine, and mequitazine. (*S*)-CAR exert therapeutic action but (*R*)-CAR is inactive.	[100]
***R/S*** **(±)-fluoxetine** **(FLX)**	Human Breast milk	FLX is administered as a racemic mixture, (*R*)-FLX and (*S*)-FLX are *N*-demethylated to (*R*)-NFLX (norfluoxetine) and (*S*)-NFLX.	LC-MS/MS Column: RAM-C_18_-BSA in the first dimension and Chirobiotic^TM^ V2 in the second dimension. Mobile phase: 10 mM aqueous AA (pH 6.8): EtOH (20:80 *v*/*v*) at 25 °C Flow rate: 0.4 mL min^−1^	(*R*)-FLX, (*S*)-FLX, and (*S*)-NFLX are equally potent selective serotonin reuptake inhibitors, while (*R*)-NFLX is 20-fold less potent.	[101]
***R/S*** **(±)-linagliptin** **(LGN)**	Human Plasma, urine, and feces	The pharmacokinetics and metabolism of LGN were investigated in healthy volunteers. Unchanged LGN was the most abundant radioactive species in all matrices investigated. The metabolite was identified as a (*S*)-3-hydroxypiperidine derivative of LGN.	LC-MS/MS Column: Chiralpak^®^ IA Mobile phase: MeOH/EtOH (1:1 *v*/*v*) 0.1% tetraethyl amine and MeOH/EtOH (1:1 *v*/*v*) with a column temperature of 30 °C. Flow rate: 0.7 mL min^−1^	LGN is a novel, orally active, highly specific, and potent inhibitor of dipeptidyl peptidase-4 that is currently used for the treatment of type 2 diabetes mellitus.	[102]

AA: Ammonium Acetate; ACN: Acetonitrile; AcOH: Acetic Acid; DAD: Diode-Array Detector; DEA: Diethylamine; ESI: Electrospray Ionization; EtOH: Ethanol; FA: Formic Acid; FD: Fluorescent Detector; HEX: Hexane; Iso: Isopropanol; MeOH: Methanol; MS/MS: Mass Spectrometry; TEA: Triethylamine; UV: Ultraviolet; 2D: Two-dimensional.
